# The conserved transmembrane proteoglycan Perdido/Kon-tiki is essential for myofibrillogenesis and sarcomeric structure in *Drosophila*

**DOI:** 10.1242/jcs.150425

**Published:** 2014-07-15

**Authors:** Juan J. Pérez-Moreno, Marcus Bischoff, Maria D. Martín-Bermudo, Beatriz Estrada

**Affiliations:** 1 Centro Andaluz de Biología del Desarrollo, Universidad Pablo de Olavide-CSIC, 41013 Seville, Spain; 2 Department of Zoology, University of Cambridge, Downing Street, Cambridge, CB2 3EJ, UK

**Keywords:** Myogenesis, Muscle, Myofibril, Sarcomere, Integrin, Chondroitin sulfate proteoglycan, Perdido, Kon-tiki, CSPG4, MCSP, AN2, NG2

## Abstract

Muscle differentiation requires the assembly of high-order structures called myofibrils, composed of sarcomeres. Even though the molecular organization of sarcomeres is well known, the mechanisms underlying myofibrillogenesis are poorly understood. It has been proposed that integrin-dependent adhesion nucleates myofibrils at the periphery of the muscle cell to sustain sarcomere assembly. Here, we report a role for the gene *perdido* (*perd*, also known as *kon-tiki*, a transmembrane chondroitin proteoglycan) in myofibrillogenesis. Expression of *perd* RNAi in muscles, prior to adult myogenesis, can induce misorientation and detachment of *Drosophila* adult abdominal muscles. In comparison to controls, *perd*-depleted muscles contain fewer myofibrils, which are localized at the cell periphery. These myofibrils are detached from each other and display a defective sarcomeric structure. Our results demonstrate that the extracellular matrix receptor Perd has a specific role in the assembly of myofibrils and in sarcomeric organization. We suggest that Perd acts downstream or in parallel to integrins to enable the connection of nascent myofibrils to the Z-bands. Our work identifies the *Drosophila* adult abdominal muscles as a model to investigate *in vivo* the mechanisms behind myofibrillogenesis.

## INTRODUCTION

The study of muscle development and maintenance is crucial for better understanding the basis of myopathies. Muscle development is a multistep process that is conserved across the animal kingdom. It starts with the specification of muscle precursor cells, the myoblasts, which either fuse to each other to form the multinucleated muscles or differentiate into cardiomyocytes. While fusion is taking place, muscles migrate and attach to tendon cells. Then, at late stages during muscle morphogenesis, final differentiation of the muscles takes place by the assembly of myofibrils (Schejter and Baylies, 2010; Schweitzer et al., 2010).

Muscles contain dozens of myofibrils, which are rod-like structures composed of the repetition of the basic functional unit of the muscle: the sarcomere. Sarcomeres are contractile units composed of interconnected thin (actin) and thick (myosin) filaments and several associated proteins, including Tropomyosin, Troponin, Titin, Zasp, CAPZ and α-actinin, among others. Myofibrillogenesis is initiated by the formation of a regular array of sarcomeres, which later on grow in width and, in some cases, in length. Contiguous sarcomeres attach to each other by the crosslinking of their thin filaments to actin-binding α-actinin at the so-called Z-disc. Thus, the Z-discs hold the sarcomeres in register as the muscles contract and prevent them from coming out of alignment when muscles stretch (Katzemich et al., 2012). Thick filaments are also crosslinked to each other in the middle of the sarcomere at the M-line. Myofibrils are connected to other components of the cell cytoskeleton and internal organelles, such as the transverse tubules, sarcoplasmic reticulum and microtubules, as well as to each other. Peripheral myofibrils are also connected to the sarcolemma (cell membrane) and to the extracellular matrix (ECM) at specialized integrin adhesion sites called costameres (Ervasti, 2003). In addition, myofibrils termini also attach to the skeleton through integrins at the so-called myotendinous junction (Sanger et al., 2005; Sparrow and Schöck, 2009).

Studies in tissue culture and genetic model organisms have shed light onto the genetic, cellular and molecular basis of myofibril assembly (Sanger et al., 2005; Sparrow and Schöck, 2009; Rui et al., 2010). There are two prominent models for myofibrillogenesis. In the ‘independent assembly model’, proposed by Holtzer and colleagues, microfilament bundles act as a scaffold during sarcomere assembly (Holtzer et al., 1997). I-Z-I complexes containing actin filaments, α-actinin and Titin are organized in register on these filamentous structures. Full-length myosin thick filaments assemble independently and incorporate in these preformed structures (Schultheiss et al., 1990). In the ‘premyofibril model’, proposed by Sanger and co-workers, premyofibrils, which contain transitory arrays of I-Z-I complexes consisting of nonmuscle myosin II and sarcomeric actin attached to precursors of Z-discs (Z-bodies) rich in α-actinin, form at the periphery. Premyofibrils develop into mature myofibrils concurrent with a replacement of non-muscle myosin II by muscle myosin II. Then, Z-bodies grow and align longitudinally in the future Z-disc through a contractility-dependent maturation. As maturation proceeds, many myofibrils are displaced to the interior while being connected to each other by the inter-Z-disk bridges (Rhee et al., 1994). In an extension of the premyofibril model, integrin adhesion sites (IAS) are proposed to be key in nucleating the first components of myofibrils (Sparrow and Schöck, 2009). In this scenario, IAS, also called protocostameres, act as nucleation sites for α-actinin recruitment, which then causes the assembly of premyofibril-associated Z-bodies. This model is based on genetic evidence from several model organisms including mice, *Drosophila* and *C. elegans* (reviewed in Sparrow and Schock, 2009). In all of them, integrins are required for sarcomere assembly and Z-disc formation. Furthermore, in *Drosophila* and *C. elegans*, integrins are the most upstream components in myofibril assembly. However, despite this crucial role for integrins in myofibrillogenesis, the mechanisms by which integrins get localized and act in coordination with other important proteins during myofibrillogenesis remains unclear.

The *Drosophila* adult muscles have proven to be an excellent model system to study the genetic, cellular and molecular basis of myogenesis (Chen and Olson, 2004; Beckett and Baylies, 2006). Morphologically, there are two major muscle types in the *Drosophila* adult: fibrillar muscles, which are present exclusively as indirect flight muscles, and tubular muscles, which include the jump, leg and abdominal muscles (Peckham et al., 1990; Schönbauer et al., 2011). In this work, we used the adult abdominal muscles as our model system to study adult myogenesis *in vivo*. The abdominal muscles are located just beneath the epidermis, so they are accessible to microscopic visualization, and are amenable to genetic analysis. Adult abdominal muscles develop *de novo* during metamorphosis from pools of myoblasts present in the larva. Proliferating myoblasts are in close contact with abdominal nerves and migrate out across the developing abdominal epidermis. This migration is followed by myoblast fusion and segregation into cell groups to form multinucleated muscle precursors. Muscle precursors migrate at both ends to find and attach to their tendon cells located in the overlying ectoderm (Currie and Bate, 1991; Dutta et al., 2005; Krzemien et al., 2012). At this point, muscle differentiation starts with the newly developed myofibers entering in the hypertrophic phase of growth, where the muscle volume increases owing to massive expression of structural genes and the assembly of the contractile apparatus.

The gene *perdido*/*Kon*-*tiki* (*perd*) is expressed in muscles and it is required for the development of the embryonic myotendinous junction. *perd* encodes a conserved single-pass transmembrane chondroitin sulfate proteoglycan, an adhesion protein ortholog of the mammalian receptor CSPG4. Perd contains laminin globular extracellular domains and a small intracellular domain with a C-terminal PDZ-binding consensus sequence (Estrada et al., 2007; Schnorrer et al., 2007). In addition, *perd* genetically interacts with integrins during embryonic myogenesis (Estrada et al., 2007). Interestingly, *perd* mutants are embryonic lethal, even though the muscle detachment phenotype affects only a subset of the embryonic muscles. In order to investigate possible additional functions of *perd* during myogenesis, we have studied its function in the context of adult fly muscles.

Here, we have identified Perd as a key regulator of myofibrillogenesis in the *Drosophila* adult abdominal muscles. We show that the expression of *perd*-specific RNA interference (RNAi) constructs in the muscles before adult myogenesis starts can induce misorientation and detachment of *Drosophila* adult abdominal muscles, a phenotype similar to the one described in the muscles of *perd* mutant embryos. In addition, *perd*-depleted muscles contain fewer myofibrils than control muscles. The few remaining myofibrils found in *perd*-depleted muscles localize at the periphery of the cell, are detached from each other and present a defective sarcomeric structure. We propose that the ECM receptor Perd has a specific role in the assembly of myofibrils and in sarcomeric organization. In our model, Perd acts downstream or in parallel to integrins to enable the connection of nascent myofibrils to the Z-bands. In addition, our work presents the *Drosophila* adult abdominal muscles as a new genetically tractable model to investigate *in vivo* the cellular and molecular mechanisms of myofibrillogenesis.

## RESULTS

### Perd is required for the development of adult muscle fibers

**Fig. 1.**
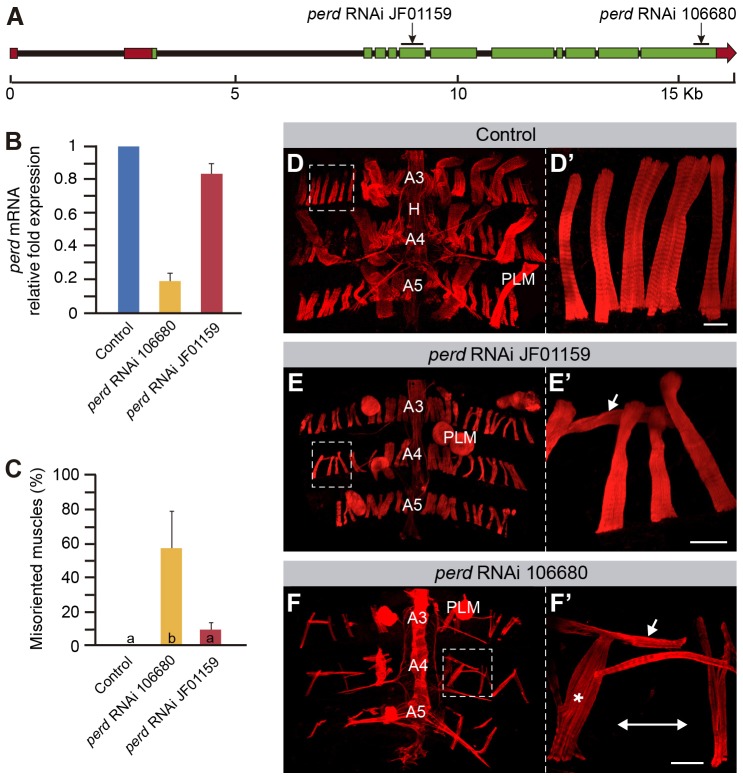
***perd* RNAi expression causes defective adult muscle development.** (A) Schematic representation of the *perd* genomic region. The sequences targeted by the two RNAi constructs used are indicated. UTR sequences are represented in red, and the exons in green. Black lines represent the introns. (B) Quantitative PCR data shows that expression of either of the two RNAi constructs results in a reduction in *perd* mRNA levels, with the 106680 construct being more efficient (means±s.d., *n* = 2). (C) Quantification of the percentage of misoriented dorsal abdominal muscles per hemisegment (means±s.d., *n* = 9). Bars labeled with different letters indicate statistically significant differences. (D–F′) Confocal micrographs of control muscles (D,D′), and muscles expressing RNAi against *perd* (E–F′) labeled with Rhodamine–Phalloidin (red). D′, E′ and F′ are magnifications of the white boxes in D, E and F, respectively. (D,D′) At 80–100 hours APF, control dorsal abdominal muscles orient parallel to the anterior–posterior axis and are homogenously distributed along the abdominal segment. (E–F′) Expression of either of the *perd* RNAi constructs results in misoriented dorsal abdominal muscles (arrows) and complete detachment of the PLM. (F,F′) In addition, expression of the RNAi 106680 construct causes abnormal distribution of the dorsal abdominal muscles with regions devoid of muscles (double arrow) and muscle bundles (asterisk). These muscles are thinner than the controls. A3, A4 and A5 indicate the corresponding abdominal segments. H, heart. Scale bars: 20 µm.

Given the role of Perd during embryonic muscle morphogenesis (Estrada et al., 2007; Schnorrer et al., 2007), we decided to analyze Perd function in adult myogenesis. To this end, we used the GAL4/UAS system to express *perd*-specific RNAi lines starting from the larval period, prior to adult abdominal myogenesis, in larval myoblasts (Bate et al., 1991; Brand and Perrimon, 1993; Dietzl et al., 2007). We utilized the Mef2-GAL4 driver to express two UAS-*perd* RNAi lines, JF01159 and 106680, which target exons present in all known *perd* isoforms (Fig. 1A; Flybase). Quantification of *perd* mRNA levels showed that, although expression of the 106680 line reduced the mRNA levels by 80.4% relative to the controls, line JF01159 was less efficient, as it decreased *perd* mRNA levels by only 17% (Fig. 1B).

Knocking down *perd* by expressing the 106680 line in the thoracic indirect flight muscles causes a strong muscle detachment phenotype and the rounding of the muscles (data not shown), similar to the phenotype in *perd* mutant embryos. The rounded shape of these muscles obstructs the study of muscle morphogenesis and subsequent differentiation, as sarcomeric proteins collapse at the center of the muscle (Rui et al., 2010). Thus, we turned to the analysis of abdominal muscles, which are less affected by *perd* knockdown and can maintain some morphological features. The dorsal abdominal musculature contains two types of muscles, the dorsal abdominal adult muscles and a set of persistent larval muscles (PLMs). Both types of muscles are oriented along the anterior–posterior axis and attach to tendon cells in the overlying epidermis (Bate et al., 1991; Krzemien et al., 2012) (Fig. 1D). We found that the expression of either of the *perd* RNAi lines in myoblasts from the larval period onwards prevented the eclosion of young adults from the pupal cage. This process requires muscle contraction, including that of the PLMs. Consistent with this, PLMs from Mef2-GAL4>UAS-*perd* RNAi abdomens (hereafter referred to as *perd*-depleted abdomens) were always detached and formed myospheres (Fig. 1E,F). This mutant phenotype was less conspicuous in the dorsal abdominal muscles, which required the co-expression of *perd* RNAi lines with Dicer to induce muscle detachment (supplementary material Fig. S1A). Thus, consistent with its role in the embryo, Perd is also required for muscle attachment in the adult. In addition to the above phenotypes, *perd*-depleted abdomens always displayed an abnormal uneven distribution of muscles, rendering abdomen areas devoid of muscles (Fig. 1F, see below). Interestingly, the adult abdominal muscles were often misoriented and thinner (Fig. 1C,E,F; supplementary material Fig. S1A). As expected, the phenotypes due to expression of line 106680 were stronger than those obtained with line JF01159 (Fig. 1E,F). Thus, from now on, we will refer only to the results obtained with line 106680. In order to validate these results we expressed the line 106680 under the adult-muscle-specific 1151-GAL4 line. These abdomens presented misoriented dorsal abdominal muscles, which were thinner than the controls and formed muscle bundles (supplementary material Fig. S1B), similar to those in Mef2-GAL4>UAS-106680 abdomens (Fig. 1F).

### Perd is required for proper adult muscle targeting and segregation

**Fig. 2.**
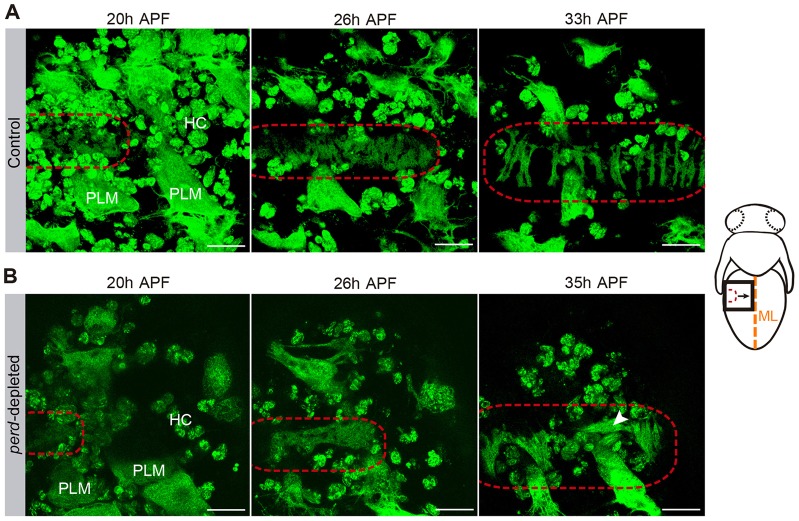
***In vivo* analysis of control and *perd*-depleted adult muscles.** Stills taken from live imaging of control adult myoblasts (A) and myoblasts expressing *perd* RNAi 106680 (B) (*n* = 7). Larval, adult abdominal muscles, and hemocytes are labeled with mCD8–GFP. Muscles of the second abdominal segment are shown. See cartoon at the right of the panel for illustration. (A) From 20 to 33 hours APF, control pupae adult myoblasts migrate, orientate along the anterior–posterior axis and separate into individual muscle fibers. (B) *perd*-depleted adult myoblasts are sometimes misoriented (arrowhead) and fail to separate properly. Red-hatched lines indicate the adult myoblasts and the forming muscle fibers. ML, midline; HC, hemocytes. Scale bars: 50 µm.

To study the muscle phenotypes of *perd*-depleted abdomens in more detail, we analyzed *in vivo* muscle migration. In control abdominal muscles, unfused adult myoblasts start migrating at 13 hours after puparium formation (APF) from lateral positions of the pupal abdomen along the dorsal nerve towards the midline (supplementary material Movie 1). This movement takes place underneath the overlying migrating epidermis. Later, myoblast fusion occurs at 20 to 26 hours APF, while migration continues. Myoblasts then coalesce into small groups that form the anlagen of individual muscle fibers and align parallel to one another in an anterior-posterior direction. At 33 hours APF most muscles are already properly segregated and oriented (Bate et al., 1991) (Fig. 2A; supplementary material Movie 1). *In vivo* analysis of myoblast migration in *perd*-depleted abdomens showed that, by 26 hours APF, some muscles failed to arrange along the anterior–posterior axis, so that by the end of myogenesis more than 50% of the muscles were abnormally oriented (Fig. 1F, Fig. 2B; supplementary material Movie 2). In addition, *perd*-depleted muscles failed to segregate from each other and arrange into muscle bundles, suggesting that the grouping of myoblast into discrete pools to individualize the different muscles is affected upon *perd* depletion (Fig. 1F; Fig. 2B; supplementary material Movie 2). Taken together, these results show that *perd* is also required in the adult to control myogenesis, including muscle attachment, orientation and segregation.

### Founder myoblast number and myoblast fusion are normal in *perd*-depleted muscles

**Fig. 3.**
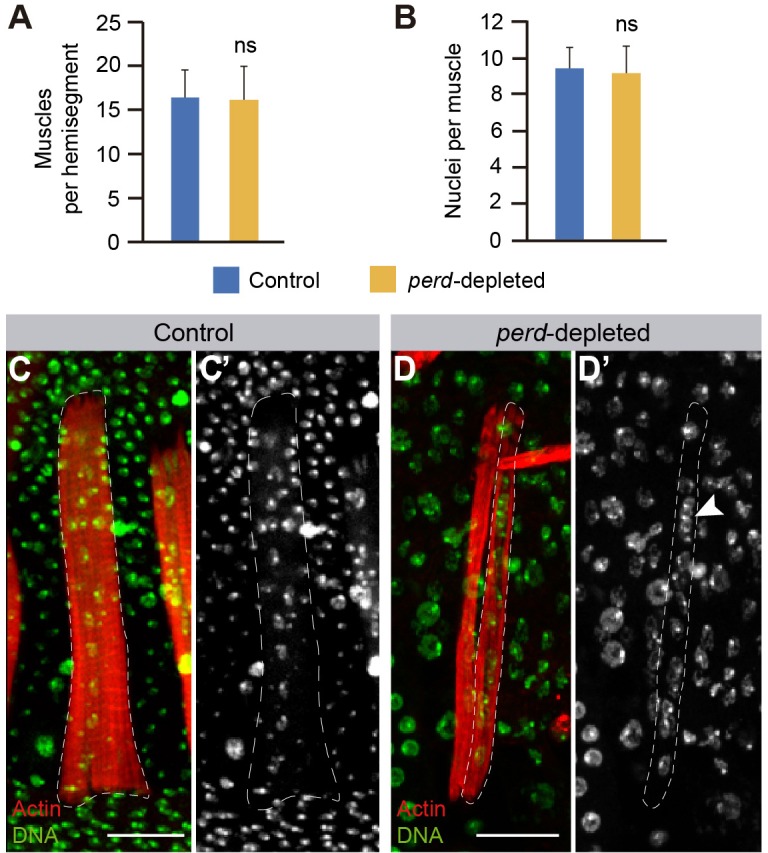
**Reduction of *perd* function does not affect muscle number or number of nuclei per muscle.** Quantification of the number of muscles per hemisegment (means±s.d., *n* = 9) (A) and nuclei per muscle (means±s.d., *n* = 9) (B) in wild-type and *perd*-depleted abdomens. ns, not significant. (C,D) Maximum intensity projection of muscles labeled with Rhodamine–Phalloidin (red) and the nuclear marker TO-PRO 3 (green). Non-muscle nuclei are also labeled with TO-PRO 3 but are located outside the muscle lumen. (D) Two *perd*-depleted muscles are shown but only the delineated one is complete and shows all its nuclei. (C) In wild-type muscles, nuclei are evenly distributed along the muscle length. This distribution is affected in *perd*-depleted muscles (arrowhead in D′). Scale bars: 20 µm.

As mentioned above, *perd*-depleted abdomens showed gaps in their muscle distribution (Fig. 1F). In order to test whether this was due to a role for *perd* in muscle specification, we quantified the number of muscles per hemi-segment in control and experimental abdomens (*n* = 9). We found that silencing *perd* in myoblasts did not affect muscle number (Fig. 3A). Furthermore, given that, like in the embryo, the number of adult abdominal muscles is directly related to the number of founder myoblasts (Dutta et al., 2005), our results suggest that *perd* is not required to specify the correct number of muscles founder cells in the adult abdomen. This is in agreement with results obtained in the embryo where *perd* is not required for founder cell specification (Artero et al., 2003; Estrada et al., 2006; Estrada et al., 2007).

*Perd*-depleted adult abdominal muscles were thinner than wild-type muscles (Figs 1, 3). In the adult fly, as it is the case during embryogenesis, muscles form by myoblast fusion. In this process, new myoblasts incorporate into growing muscles allowing them to reach their final size. Thus, the reduced muscle width observed in *perd*-depleted muscles could be due to defective myoblast fusion. To test this possibility, we quantified the number of nuclei per muscle in both wild-type and *perd*-depleted abdomens. We found that wild-type and *perd*-depleted adult abdominal muscles contained the same number of nuclei, which on average was between eight and eleven (Fig. 3B–D) (Mukherjee et al., 2011). These results show that the abnormal size of *perd*-depleted muscles is not due to fusion defects. However, it is worth mentioning here that, although in wild-type abdominal muscles the nuclei were evenly distributed along the muscle length, this was not the case in *perd*-depleted muscles (Fig. 3D). This could reflect an abnormal organization of the muscle cytoskeleton and/or organelles (see below).

### Perd is required for myofibril assembly

**Fig. 4.**
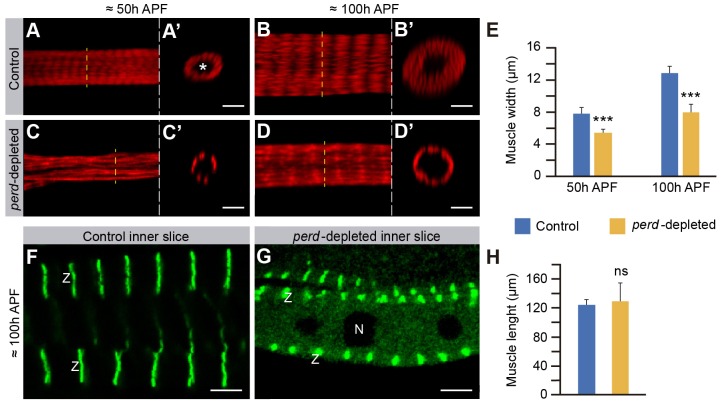
**Myofibril assembly is disrupted in *perd*-depleted muscles.** (A–D′) Confocal micrographs of muscles at 50 and 100 hours APF stained with Rhodamine–Phalloidin (red). A′–D′ show orthogonal confocal cross sections of the muscles in A–D, respectively. The asterisk marks the muscle lumen. (A–B′) From 50 to 100 hours APF, control muscles widen by increasing the amount of myofibrils. (C–D′) *perd*-depleted muscles are thinner than control at both 50 and 100 hours APF. (E) Quantification of the muscle width of both control and *perd*-depleted muscles at 50 and 100 hours APF (means±s.d., *n* = 27). Note the *perd*-depleted muscles grow very little compared to control muscles. (F,G) Longitudinal confocal cross sections of the muscles, at 100 hours APF, expressing Zasp–GFP (green) to label the Z-bands (Z). (F) In control muscles, Zasp–GFP is found in bands spanning the fiber width from the sarcolemma to the muscle lumen. (G) These bands are thicker and shorter in *perd*-depleted muscles. In addition, some Zasp–GFP can be seen in the cytoplasm surrounding the nuclei (N). (H) Quantification of the muscle length reveals that *perd*-depleted muscles are as long as controls (means±s.d., *n* = 27). ns, not significant; ***P<0.001. Scale bars: 5 µm.

To identify the cause for the thinner appearance of *perd*-depleted muscles, we measured muscle width at two time points of their development, 50 and 100 hours APF. To this end, we stained abdomens with Phalloidin to visualize filamentous actin, and analyzed orthogonal optical sections of abdominal muscles. We found that the width of wild-type abdominal muscles at 50 hours APF was on average 7.8 µm, whereas at 100 hours APF it was 12.8 µm (Fig. 4A,B,E), demonstrating that, even though the abdominal muscle pattern at 50 hours APF was completed (Bate et al., 1991), muscles sustain significant growth during late pupal development. Tubular muscles contain an internal lumen devoid of actin where nuclei are found (Peckham et al., 1990; Schönbauer et al., 2011). Our studies showed that the lumen was maintained constant in size along development (supplementary material Fig. S2), suggesting that the increase in width found in wild-type muscles was due to an increment in myofibrils and not in lumen diameter (Fig. 4A′,B′). Quantification of *perd*-depleted muscles showed that they were thinner than wild-type muscles, as their width at 50 and 100 hours APF was 5.5 and 7.9 µm, respectively (Fig. 4C–E). Interestingly, *perd*-depleted muscles increased their diameter at a much lower rate than the control ones, as the width of control muscles grows from 50 hours APF to 100 hours APF by 5 µm, whereas that of *perd*-depleted muscles only grew by 2.4 µm. In addition, the lumen perimeter of *perd*-depleted muscles was slightly but significantly larger than the controls at 100 hours APF (supplementary material Fig. S2). Our results strongly suggest that *perd*-depleted muscles are thinner because they contain fewer myofibrils than controls, pointing towards a role for *perd* in adult myofibrillogenesis. To further test the role of Perd in myofibrillogenesis, we looked at the pattern of expression of Zasp–GFP (a protein-trap inserted in the genomic locus of the Z-disc-associated protein Zasp66), which has been extensively used to visualize muscle structure (Jani and Schöck, 2007). In wild-type dorsal abdominal muscles, Zasp–GFP showed a transversal banding distribution, reflecting the perfect alignment of sarcomeres from different myofibrils (Fig. 4F). Each band spanned the entire fiber width, from the sarcolemma to the muscle lumen, which was devoid of Zasp–GFP expression (Fig. 4F). Analysis of Zasp–GFP expression in *perd*-depleted muscles showed that, even though the transverse banding pattern was preserved, the bands were thicker and shorter, and were confined to the periphery of the fiber (Fig. 4G). This suggests that the only differentiated myofibrils with a sarcomeric organization present in these muscles were positioned at the periphery of the muscle, close to the sarcolemma. In addition, we observed that Zasp–GFP was present not only in the Z-bands of *perd*-depleted muscles but also in the cytoplasm surrounding their nuclei. This could represent a pool of excess protein owing to a reduced number of assembled myofibrils (Fig. 4G). Finally, despite the width differences, the length of *perd*-depleted muscles was similar to controls (Fig. 4H), showing that *perd* is not required for myofibril elongation. In summary, our results show that Perd is required for myofibril organization and differentiation, and thus it is essential for the proper growth of adult abdominal muscles.

### Perd is required for sarcomeric organization

**Fig. 5.**
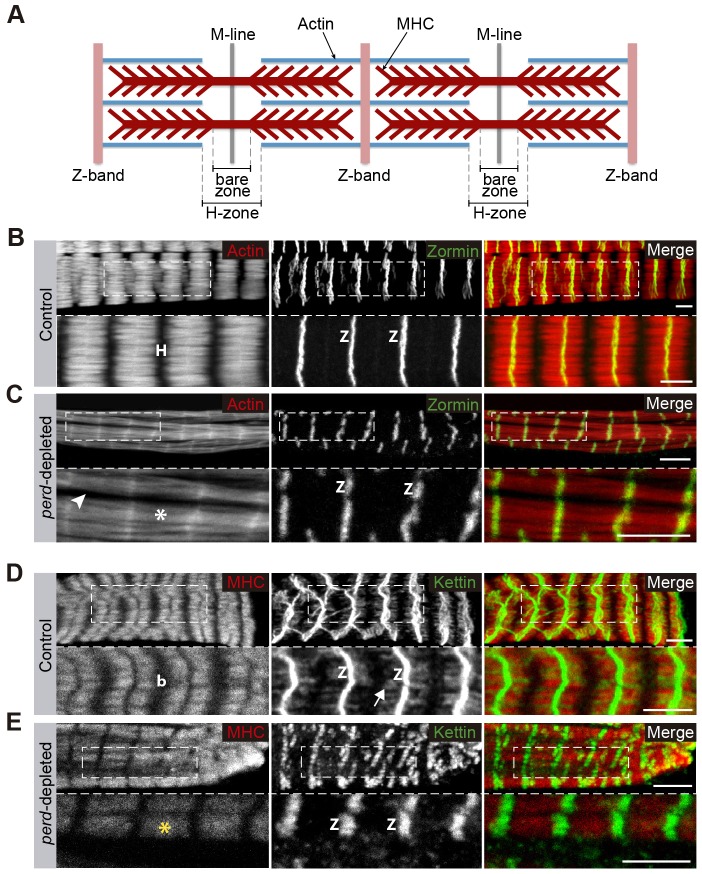
**Sarcomeric structure is affected in *perd*-depleted muscles.** (A) Schematic diagram of the sarcomeric structure. (B–E) Confocal micrographs of control (B,D) and *perd*-depleted muscles (C,E). (B,C) Rhodamine–Phalloidin (red) and anti-Zormin antibody (green). In control muscles, the Z-band protein Zormin forms straight lines (B); these are discontinuous and irregular in *perd*-depleted muscles (C). In addition, the H-zone (H), labeled by the absence of Phalloidin in controls (B), is missing in *perd*-depleted muscles (white asterisk in C), Phalloidin staining also shows gaps between myofibrils (arrowhead in C). (D) In control muscles, Kettin is found at the Z-bands and extends towards the central part of the sarcomere (arrow). (E) However, in *perd*-depleted muscles, Kettin is restricted at the Z-bands. In addition, MHC staining (red) reveals that the bare zone (b), contained within the H-zone, is also missing in *perd*-depleted muscles (yellow asterisk). To facilitate sarcomere visualization, the magnified regions are single slices. Z, Z-band. Scale bars: 5 µm.

The banding distribution of Zasp–GFP in *perd*-depleted abdominal muscles might suggest that *perd* is not absolutely required for sarcomeric organization. To further test this, we analyzed in more detail the distribution of other structures and proteins present in mature sarcomeres in the few myofibrils present in *perd*-depleted muscles. We examined the expression of the Z-disc proteins Zormin and Kettin, and the disposition of the thin and thick filaments. In wild-type abdominal muscles, Zormin and Kettin formed straight lines, owing to the proper alignment and attachment of myofibrils at the Z-bands (Fig. 5B,D) (Bullard et al., 2005; Hudson et al., 2008). In contrast, the Zormin- and Kettin-positive lines in *perd*-depleted muscles were irregular and discontinued (Fig. 5C,E), reflecting a role for Perd in the alignment and attachment between myofibrils. In addition, although Kettin expression extended at both sides of the Z-bands in wild-type muscles, this did not occur in *perd*-depleted muscles (Fig. 5D,E). To visualize the thin and thick filaments, we used Phalloidin and antibodies against myosin heavy chain (MHC), respectively. In wild-type muscles, Phalloidin labeled the sarcomeric actin of the thin filaments, thus highlighting the H-zone that appeared as a region devoid of Phalloidin (Fig. 5A,B). Phalloidin staining of *perd*-depleted muscles showed that the H-zone was absent or very much reduced in these muscles compared to control (Fig. 5C). This was supported by the results obtained from the analysis of MHC distribution in wild-type and *perd*-depleted muscles. In wild-type sarcomeres, there were two bands devoid of MHC staining, a narrow one corresponding to the Z-band and a wider one, known as the bare zone (Tskhovrebova and Trinick, 2003) located within the H-zone (Fig. 5D). In agreement with the results from the Phalloidin staining, we found that the bare zone was absent in *perd*-depleted muscles (Fig. 5E). Overall, the analysis of different sarcomeric proteins shows that Perd is required for the proper organization of the sarcomeric structure.

### Perd function in myofibrillogenesis is independent of its function in myotendinous junction development

**Fig. 6.**
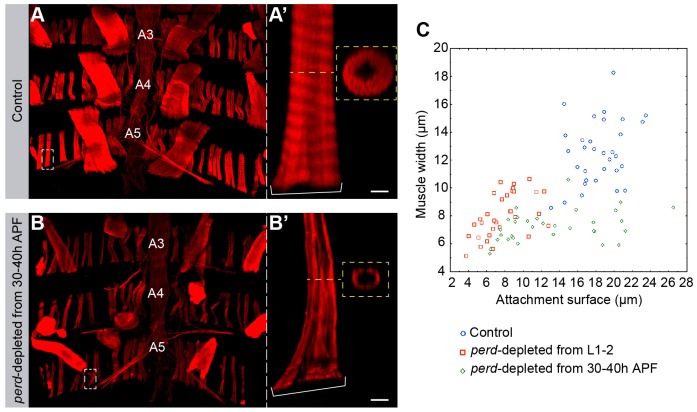
**An independent role for Perd function in myofibrillogenesis.** (A–B′) Confocal micrographs of muscles stained with Rhodamine–Phalloidin (red) at the end of pupal development. Dotted lines and white brackets indicate muscle width and attachment surface, respectively. (A) Control dorsal abdominal muscles. (A′) Magnification of a control muscle showing its muscle attachment surface and its width, which can also be seen in the cross section in A′. (B) *perd*-depleted abdomens, where RNAi was induced starting at 30–40 hours APF, once the muscle migration has finished and the muscle attachments have been established. (B′) Magnification of a *perd*-depleted muscle. Note that this muscle is thinner even though it has an attachment surface similar to the control. A cross-section of this muscle shows that it contains less myofibrils than the control. (C) Scatter plot of muscle widths (*y*-axis) in relation to their attachment surface (*x*-axis) in control muscles, in muscles where *perd*-RNAi was expressed starting from larval (L) period 1 to 2, and in muscles where *perd*-RNAi was expressed starting from 30 to 40 h APF. A3, A4 and A5 indicate the corresponding abdominal segments. Scale bars: 5 µm.

Myogenesis is a multistep process where muscle attachment occurs prior to myofibrillogenesis (Sparrow and Schöck, 2009). As many *perd*-depleted muscles showed a reduced attachment area (Fig. 1D′,F′; Fig. 3C,D), it is possible that the defects found in myofibrillogenesis were a consequence of aberrant muscle attachment. To test this, we reduced the levels of Perd after muscle attachment was established and analyzed myofibril content in *perd*-depleted muscles that had normal attachment sites. To test this, we expressed *perd* RNAi starting at 30 to 40 hours APF, by which time muscle targeting and attachment have already occurred (Bate et al., 1991). Indeed, this treatment resulted in *perd*-depleted muscles in which the length of the attachment surface was similar to that in control muscles (Fig. 6). Interestingly, we found that the myofibril content, as measured by muscle width, was still compromised in these muscles (Fig. 6B′,C). This result suggests that the role of Perd in myofibrillogenesis is independent of its role in muscle attachment sites.

### Perd localizes to the muscle tendon attachment and the costameres in adult abdominal muscles

**Fig. 7.**
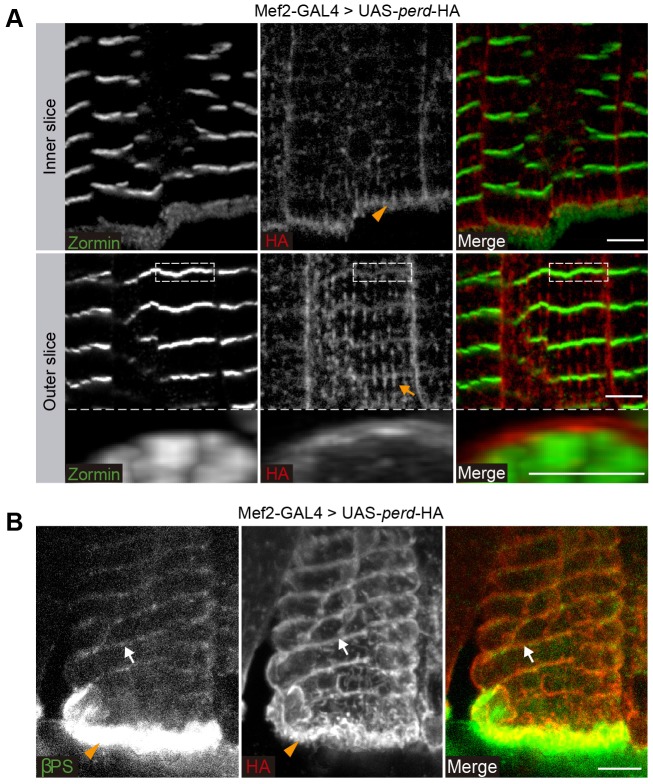
**Perd localizes at muscle attachment sites and costameres.** (A) Confocal micrographs of muscles expressing an HA-tagged Perd protein (tub-GAL80ts/+; Mef2-GAL4/UAS-*perd*-HA) stained for HA (red) and Zormin (green). Inner slice: an internal cross-section of these muscles reveals that, although Zormin localizes to Z-bands, HA-tagged Perd is mainly found at muscle attachment sites (orange arrowhead). Outer slice: an external cross-section showing that HA-tagged Perd also localizes in discrete circumferential bands that juxtapose the Zormin Z-bands (white dotted rectangle). Orthogonal cross section of this region (white dotted rectangle) showing that Perd surrounds the myofibrils. Perd is also found in perpendicular stripes to the Z-bands, parallel to the longitudinal axis of the muscle (orange arrow). (B) Confocal micrographs of muscles expressing HA-tagged Perd protein stained for HA (red) and βPS integrin (green). HA-tagged Perd colocalizes with βPS at both the attachment site (orange arrowhead) and the costameres (white arrow). Scale bars: 5 µm.

To help us to understand the role of Perd in myofibrillogenesis and sarcomeric organization during adult myogenesis, we studied its expression pattern in adult abdominal muscles. Antibodies against the Perd protein have revealed that Perd localizes at the myotendinous junction in embryonic muscles (Schnorrer et al., 2007). Unfortunately, our attempts to examine the expression of Perd in adult muscles using these antibodies proved inconclusive owing to inconsistent results and to the weakness of the signal. To circumvent this problem, we used a Perd–HA fusion construct that has been shown to rescue Perd embryonic function (UAS-*kon*-HA) (Schnorrer et al., 2007). When we expressed Perd–HA in adult muscles with the Mef2-Gal4 driver, we found that, in addition to its localization to the myotendinous junction (Fig. 7A), it localized in the sarcolemma in discrete circumferential bands distributed along the muscle length (Fig. 7A). This banding expression in the sarcolemma is in register with the Zormin Z-bands from the underlying myofibrils (Fig. 7A).

Costameres have been suggested to function in the lateral transmission of contractile forces from sarcomeres across the sarcolemma to the ECM (Ervasti, 2003). For this reason, and taking into account the fact that many canonical focal adhesion proteins, such as integrins, Talin, α-actinin and Vinculin, are found in costameres, it has been proposed that costameres are analogous to the focal adhesions found in non-muscle cells (Sparrow and Schöck, 2009). In *Drosophila*, integrins localize to costameres in the adult ventral abdominal muscles (Ribeiro et al., 2011). In order to determine whether Perd–HA localizes to the costameres, we have performed colocalization studies with Perd–HA and integrins. We found that the circumferential expression of Perd–HA indeed colocalized with the costameric marker βPS integrin (also known as Myospheroid) (Fig. 7B). Furthermore, genetic studies in *Drosophila* and in *C. elegans* have highlighted a key role for integrins in myofibril assembly (Volk et al., 1990; Hresko et al., 1994; Bloor and Brown, 1998). Thus, we next decided to test possible interactions between *perd* and integrins.

### Perd can function independently of integrins

**Fig. 8.**
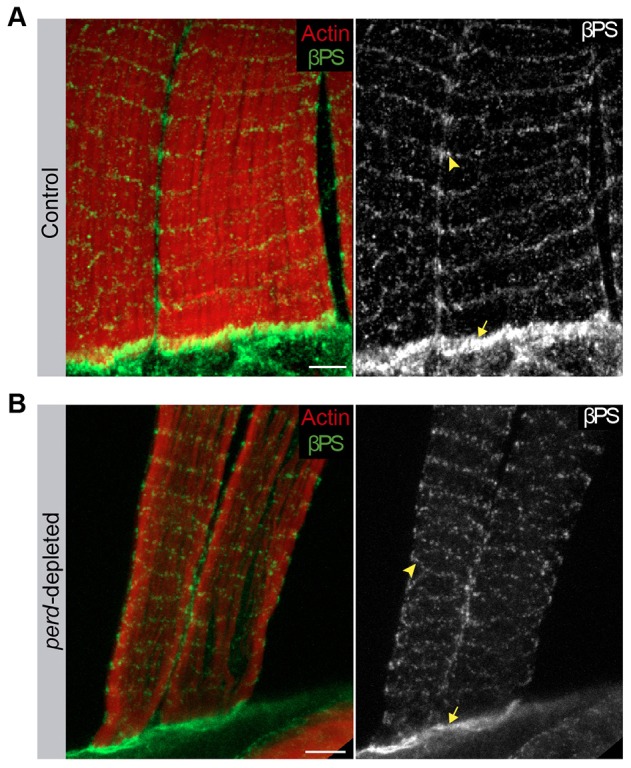
**Integrins localize properly in *perd*-depleted muscles.** (A,B) Confocal micrographs of control (A) and *perd*-depleted muscles (B) stained for Rhodamine–Phalloidin (red) and βPS integrin (green). (A) In control muscles, the βPS integrin localizes at the costameres (arrowhead) and at muscle attachment sites (arrow). (B) This localization is not affected in *perd*-depleted muscles. Scale bars: 5 µm.

A series of experiments have highlighted a role for integrins in myofibrillogenesis in *Drosophila*. Cell culture experiments using myotubes derived from *Drosophila* embryonic gastrula cells have shown that βPS integrin localizes to the Z-bands. In addition, myotubes derived from βPS mutant embryos undergo fusion but fail to form stable Z-bands (Volk et al., 1990). Furthermore, ultrastructural analysis of somatic and hindgut muscles has revealed that βPS mutant muscles lack defined Z-bands (Volk et al., 1990). Finally, muscles from embryos carrying an amorphic mutation in the αPS2 integrin (also known as Inflated) do not contain intervening H-zones (Bloor and Brown, 1998). Thus, as some of the integrin phenotypes resembled those here described for *perd*, one possible mechanism by which Perd could regulate proper myofibrillogenesis and sarcomeric organization is through the regulation of integrin localization. To test this hypothesis, we analyzed whether integrins were properly localized in *perd*-depleted muscles. As was the case for the abdominal PLMs, βPS localized at the muscle-attachment sites and at costameres in wild-type adult dorsal abdominal muscles (Fig. 8A) (Ribeiro et al., 2011). This localization was not affected in *perd*-depleted muscles (Fig. 8B). Furthermore, Talin, a core component of integrin adhesion sites, which is also found at muscle attachment sites and Z-bands, localized normally in *perd*-depleted muscles (supplementary material Fig. S3). These results showed that Perd was not required for proper localization of focal adhesion proteins at muscle attachment sites and costameres, and suggests that Perd might function independently of integrins during adult myofibrillogenesis.

## DISCUSSION

The universal nature of myogenesis suggests that common molecular mechanisms underlie this biological process. Myogenesis leading to the formation of the *Drosophila* adult muscles provides a promising, yet still quite unexplored, system in which conserved molecules regulating this process can be identified and characterized. Here, we present the adult abdominal muscles as an ideal model system to unravel new mechanisms underlying muscle morphogenesis in general, and myofibrillogenesis in particular. Using this system, we have studied the role of Perd, a conserved ECM receptor, in adult muscles. We show that, as it is the case in the embryo, Perd is required for muscle guidance and attachment in adult abdominal muscles. In addition, our results uncover a new function for Perd in myofibrillogenesis and sarcomeric organization.

A major finding of our study is that Perd performs an essential role during adult fly myogenesis, which is crucial for the growth of muscle fibers through myofibril assembly. This requirement is highly specific to the myobrillogenesis process, as other features in adult myoblast development, such as myoblast fusion or specification of founder cells, appear unaffected when the levels of *perd* are reduced. In addition, although Perd is required for muscles to properly attach to tendon cells, another key step during myogenesis, we show here that the role of Perd in myofibrillogenesis is independent of its role in muscle attachment. It is interesting to note that Perd muscle detachment can affect all dorsal adult muscles and only affects a subset of the embryonic muscles (Estrada et al., 2007; Schnorrer et al., 2007). We hypothesize that this could be owing to the fact that all dorsal abdominal muscles from a segment are morphologically and functionally similar. In contrast, each embryonic muscle is unique in its morphology, size, attachment sites and innervation, and might have different requirements for the gene *perd*.

Studies in mice, *Drosophila* and *C. elegans* have highlighted an essential role for integrins in myofibril assembly *in vivo* by regulating sarcomeric assembly and Z-disc formation. Furthermore, integrins have been proposed to act as starting points for sarcomere assembly by initiating the assembly of actin filaments at the muscle membrane (Sparrow and Schöck, 2009). However, analysis of sarcomere organization in *Drosophila* embryos lacking integrins, have shown that the association of actin and α-actinin can occur in the absence of integrins. This has led to the proposal of a new model for integrin function in myofibrillogenesis in which integrins and other protein complexes, such as Zipper, Zasp, α-actinin, the I-Z-I complex, troponin–tropomyosin and MHC, assemble independently prior to sarcomere formation. Subsequently, integrins act as anchor points for the floating I-Z-I complex and provide tension that allows the interdigitation of thin and thick filaments for *de novo* sarcomere assembly. This, in turn, might facilitate the localization of integrins on the cell membrane with a given periodicity (Rui et al., 2010). We show here that Perd localizes at the sarcolemma at the level of the Z-bands and that integrins and associated proteins, such as Talin, localized correctly in *perd*-depleted muscles. In addition, we show that initial myofibrillogenesis takes place in these muscles. From these results, we propose that Perd acts once integrins have anchored the initial sarcomeric protein complexes to the surface of the muscles. In this scenario, Perd could be required, downstream or in parallel to integrins, for either the correct assembly of the sarcomeric complex or/and for proper interactions between the recruited sarcomeric proteins (see below). This, in turn, would be necessary for the incorporation and assembly of newly formed myofibrils at the Z-discs, and for subsequent growth of the muscle fiber. This is consistent with our data showing that *perd*-depleted muscles are thinner than wild-type muscles, appear frayed and present gaps between myofibrils. In addition, we propose that Perd function in the assembly of a functional sarcomeric complex at Z-discs might also be required for correct sarcomeric organization. This is supported by our data demonstrating that diminished Perd levels results in sarcomere disorganization, as it is the case when different sarcomeric components, such as Zasp, α-actinin or Zipper, are reduced. Furthermore, reduction of integrin levels, while mantaining muscle attachment, causes the absence of H-zone similar to *perd*-depleted muscles (Bloor and Brown, 1998; Perkins et al., 2010; Rui et al., 2010). The interaction of Perd with the ECM at the Z-discs could enhance the tension transmitted through integrins that is necessary for subsequent interdigitation of thin and thick filaments. This could explain why lack of Perd results in abnormal sarcomeric banding and absence of the H-zone. Taken together, our results suggest a key role for Perd, after the initial steps mediated by integrins, which is essential for correct myofibrillogenesis and sarcomeric organization. However, we cannot completely rule out the fact that the initial myofibrillogenesis that takes place in *perd*-depleted muscles could be due to residual levels of Perd, and not just to the function of other important nucleating molecules, such as integrins. Thus, it remains possible that the role of Perd during adult myogenesis could be even more crucial and Perd, as is the case for integrins, might also be required for the initial steps of myofibril assembly.

Interestingly, we have found a genetic interaction between *perd* and integrins when simultaneously expressing the *perd* and *mys* RNAis. We found that both muscle orientation and attachment phenotypes were considerably increased when expressing simultaneously both RNAis compared to the expression of each RNAi alone. Although, the strong detachment phenotype due to the expression of both RNAis did not allow us to assess the sarcomeric structure, these results show that there is a genetic interaction between *perd* and integrins during the process of adult muscle myogenesis (supplementary material Fig. S4).

How could Perd regulate myofibrillogenesis and sarcomeric organization at the molecular level? Perd is a conserved chondroitin sulfate transmembrane proteoglycan that contains two extracellular laminin G domains and a small intracellular domain with a PDZ-binding consensus sequence, which serves as a linkage to PDZ protein networks. Interestingly, some of the proteins present at the Z-discs that are essential for Z-disc formation and myofibril assembly, such as Zasp52, Zasp66 and Zasp67 proteins, also contain a PDZ domain (Katzemich et al., 2011; Katzemich et al., 2013). Thus, Perd might function by regulating the localization of these PDZ-containing proteins. Here, we have tested the localization of Zasp66 in *perd*-depleted muscles. We found that even though it localizes to the Z-bands of the few remaining myofibrils, there is a pool of cytoplasmic Zasp66 that is not observed in wild-type muscles. Thus, Perd could be required for proper recruitment of Zasp66 to the Z-discs, which in turn might regulate the assembly of new forming myofibrils and sarcomeric organization. Yet, we cannot differentiate whether the abnormal cytoplasmic levels of Zasp66 observed upon reduction of *perd* levels are a cause or an effect of defective myofibril assembly. In an alternative scenario, the interaction of Perd with sarcomeric PDZ containing proteins might not regulate directly their localization but it could interfere with their ability to interact with other sarcomeric proteins, thus compromising their function. Our results also show that Kettin, a *Drosophila* isoform of Titin, is not properly localized in *perd*-depleted muscles. As it is the case for vertebrate Titin isoforms, Kettin binds to α-actinin, actin and other proteins at the Z-disc through its N-terminus domain, while its long polypeptide chain exits the Z-disc and transverse the I-band to bind to the distal part of the thick filament (Machado and Andrew, 2000; Linke, 2008; Sparrow and Schöck, 2009). Here, we show that in *perd*-depleted muscles the H-zone seems to be absent. In addition, we find that while Kettin localizes properly at the Z-band it fails to extend toward the middle of the sarcomere. Thus, one way to explain the absence of the H-zone in *perd*-depleted muscles is that Perd could be required for the proper localization of Kettin along the thick filament, which in turn is required for proper filament interdigitation, and therefore the formation of the H-zone. However, it is difficult to elucidate whether the abnormal distribution of Kettin observed after the reduction of *perd* levels is an effect of defective sarcomeric architecture. In the future, it will be interesting to test, at both biochemical and genetic levels, the putative interactions between Perd and other proteins localized at the Z-discs. This will allow us to establish functional relationships and hierarchies between Perd and other sarcomeric proteins during myofibrillogenesis.

The identification and functional characterization of genes required for myofibril development and maintenance is key not only for our understanding of the myogenesis process but also for the diagnosis and treatment of some muscle diseases. Here, we have identified a new specific function for the gene *perd* in the assembly of myofibrils. We propose that Perd mediates the connection between the ECM and the structural myofibril components enabling the assembly of myofibrils. The function of Perd orthologs in rats, mice and humans (CSPG4, also known as NG2, AN2 and MCSP) has been mainly studied in glia and melanocytes, where they are required for cell proliferation, migration and adhesion. Interestingly, however, CSPG4 expression has also been observed in the sarcolemma and in the neuromuscular junction of human postnatal skeletal muscle, as well as in regenerating myofibers (Petrini et al., 2003). Moreover, CSPG4 is a marker of human pericytes, a type of myogenic precursors (Dellavalle et al., 2007; Meng et al., 2011). The expression of CSPG4 in muscle cells together with the fact that it is upregulated in Duchenne muscular dystrophy, and downregulated in merosin-deficient congenital muscular dystrophy (MDC1A) muscles, respectively (Petrini et al., 2003), suggest that Perd function in myofibrillogenesis might be conserved. The myriads of tools available to reduce the levels of a particular gene in a specific moment of development in other organisms, such as mice, *C. elegans* or zebrafish, will allow us to test this hypothesis in the near future.

## MATERIALS AND METHODS

### *Drosophila* strains and genetics

Strain *y*^1^*w*^118^ was used as control. The following stocks were used: tub-GAL80ts (7108), UAS-*Dicer* (24651) and UAS-*perd* RNAi JF01159 (31584) from Bloomington *Drosophila* Stock Center (http://flystocks.bio.indiana.edu/). Kettin-GFP (ZCL2144) from FlyTrap (http://flytrap.med.yale.edu/). UAS-*perd* RNAi (106680) and UAS-*mys* RNAi (29619) from Vienna *Drosophila* RNAi Center (http://stockcenter.vdrc.at). Zasp-GFP (110740) from *Drosophila* Genetic Resource Center (http://www.dgrc.kit.ac.jp/). Mef2-GAL4 (Ranganayakulu et al., 1996). UAS-*mCD8*-GFP (Lee and Luo, 1999). UAS-*rhea*-mCherry (Venken et al., 2011). UAS-*kon*-HA (Schnorrer et al., 2007). 1151-GAL4 was a gift from Lingadahalli S. Shashidhara (Centre for Celular and Molecular Biology, Hyderabad, India). For transient expression assays, the different UAS lines were crossed with tub-GAL80ts; Mef2-GAL4 at 18°C, and the UAS expression was induced switching to 29°C. The temperature shift was made when larvae were between the first and the second larval stage, unless otherwise specified.

### *In vivo* imaging of abdominal muscles

Pupae were staged as previously described (Bainbridge and Bownes, 1981). Pupae were filmed through a window in the pupal case as previously described (Bischoff and Cseresnyés, 2009). We focused our analysis on the dorsal side (tergite) of abdominal segment A2. All wild-type imaged flies developed into pharate adults and hatched. *Z*-stacks of around 70 µm with a step size of 2.5 or 3.0 µm were recorded every 150 or 180 s at 23±2°C using a Leica SP5 confocal microscope. Figures and videos were made using Adobe Illustrator and ImageJ (NIH, Bethesda).

### Immunohistochemistry and microscopy

Pupae were cut longitudinally at 80–100 hours APF, unless otherwise specified. The dorsal parts of the abdomen were dissected in PBS, where the internal organs were gently removed. The dissected tissues were fixed in 4% paraformaldehyde in PBT (PBS containing 0.2% Triton X-100) for 20 minutes, washed in PBT for 30 minutes, and incubated with primary antibodies overnight at 4°C. Subsequently, the dorsal parts of the abdomen were washed, incubated with secondary antibodies for 1 hour at room temperature, washed again for 30 minutes, and mounted in Vectashield (Vector Laboratories). The primary antibodies used were: rabbit anti-Zormin, 1∶200 (gift from Belinda Bullard, University of York, UK); rabbit anti-GFP, 1∶400 (Life Technologies); rat anti-RFP, 1∶200 (Chromotek); rat anti-HA High Affinity, 1∶400 (Roche); rabbit anti-HA, 1∶1000 (Abcam); mouse anti-βPS, 1∶100 (DSHB); and rat anti-MHC 1∶400 (Babraham Bioscience Technologies). The secondary antibodies used were: goat anti-rabbit conjugated to Alexa Fluor 488, 1∶200, goat anti-rat conjugated to Cy3 and goat anti-rat conjugated to Cy5, 1∶200 (Life Technologies). Stainings with Rhodamine–Phalloidin, 1∶1000 (Biotium) or Alexa-Fluor-488–Phalloidin, 1∶1000 (Life Technologies) were made by incubating abdomens for 30 minutes at room temperature after secondary antibody incubation. The DNA dye TO-PRO 3 1∶1000 (Molecular Probes, Life Technologies) was incubated for 10 minutes and washed just before mounting the samples. Confocal images were obtained using a Leica SP2 microscope and processed with ImageJ (NIH, Bethesda) and Adobe Photoshop. All the images shown are maximum instensity projections, unless otherwise specified.

### Data analysis

In order to quantify the experimental phenotypes, we considered muscles to be misoriented when they showed a 45°-angle or greater with respect to the anterior–posterior axis of the abdominal segment. Moreover, we considered muscles as being detached when they presented a spindle or myospheroid shape. We obtained width measurements in the central region of each muscle.

We statistically compared differences in the variables of interest among the different genotypes. Because variables often did not meet parametric assumptions (i.e. did not show an underlying Gaussian error distribution or presented heteroscedasticity), we used non-parametric Mann–Whitney–Wilcoxon tests where only two genotypes were compared. Data on proportion of misoriented or detached muscles were compared across genotypes fitting a generalized linear model with an underlying binomial error distribution and a logit link function. Statistical analyses were conducted using the R package (http://www.r-project.org/). Results from quantification of muscle phenotypes are shown on the figures so that bars indicate means and error bars indicate standard deviation. Different letters indicate statistical differences among genotypes so that bars marked with different letters are statistically different whereas bars marked with the same letter are not.

### Quantitative PCR

*y*^1^*w*^118^ (control), *perd* RNAi JF01159 and *perd* RNAi 106680 males were crossed with Mef2-GAL4 females at 18°C and switched to 29°C when larvae were between the first and the second larval stage. TRIzol reagent (Invitrogen) was used to isolate total RNA from late pupae. Subsequently, the total RNA was treated with RQ1 RNase-Free DNase (Promega). The DNA-free RNA was used to synthesize cDNA using SuperScript III First-Strand Synthesis Supermix (Invitrogen). qPCR was performed in a Mx3000P thermocycler (Stratagene) using Power SYBR Green Master Mix (Applied Biosystems). Changes of the relative expression levels were determined by using the 2^−ΔΔCT^ method (Livak and Schmittgen, 2001). Levels of Perd mRNA were normalized to RPL32 mRNA. Primer pairs: 5′-CCGCCAACAAATCCACTACT-3′ and 5′-ATCGAATTGGAAACGCTTGT-3′ for Perd; 5′-GCAAGCCCAAGGGTATCGA-3′ and 5′-CGATGTTGGGCATCAGATACTG-3′ for RPL32.

## Supplementary Material

Supplementary Material
