# Supplementary material for: The conserved transmembrane proteoglycan Perdido/Kon-tiki is essential for myofibrillogenesis and sarcomeric structure in Drosophila
Source: J Cell Sci. 2014 Jul 15;127(14):3162–73. doi: 10.1242/jcs.150425 (PMC4095857; doi:10.1242/jcs.150425)
Supplement: Supplementary Material [file supp_127_14_3162__index.html]

The conserved transmembrane proteoglycan Perdido/Kon-tiki is essential for myofibrillogenesis and sarcomeric structure in Drosophila — Supplementary Material 

# The conserved transmembrane proteoglycan Perdido/Kon-tiki is essential for myofibrillogenesis and sarcomeric structure in *Drosophila*

## JCS150425 Supplementary Material

**Files in this Data Supplement:**

- **Supplementary Material**
