# Supplementary material for: The conserved transmembrane proteoglycan Perdido/Kon-tiki is essential for myofibrillogenesis and sarcomeric structure in Drosophila
Source: J Cell Sci. 2014 Jul 15;127(14):3162–73. doi: 10.1242/jcs.150425 (PMC4095857; doi:10.1242/jcs.150425)
Supplement: Supplementary Material [file supp_127.14.3162_JCS150425.pdf]

Supplementary Fig. 1. Co-expression of Dicer with the *perd* RNAi construct JF01159 mimics the effects of the expression of the *perd* RNAi construct 106680. Expression of 106680 driven by 1151-GAL4 mimics the effects of its expression with Mef2-GAL4 line.

(A-B') Confocal micrographs of muscles expressing RNAi constructs against *perd* stained for Rhodamine-Phalloidin (red). **(A-A'')** Muscles co-expressing the RNAi JF01159 construct against *perd* and Dicer (UAS-*Dicer*/+; Mef2-GAL4/UAS-*perd* RNAi JF01159) mimic the phenotype due to expression of the RNAi 106680 (see **Fig. 1F**). **(A', A'')** Magnifications of the white box in **A**. Arrow points to a misoriented muscle. Note that an increase in the efficiency of the RNAi also leads to a detachment of the dorsal abdominal muscles (arrowhead in **A''**). (**B, B')** The expression of the RNAi 106680 construct against *perd* with the adult muscle specific 1151-GAL4 line (1151-GAL4/Y; UAS-*perd* RNAi 106680/+) leads to misoriented dorsal abdominal muscles (arrow in **B'**), which form muscle bundles (asterisk in **B'**), and are thinner than the controls (see **Fig. 1D**). A3, A4 and A5 indicate the corresponding abdominal segments. PLM: Persistent Larval Muscle. Scale bars: 20 μm.

Supplementary Fig. 2. Muscle lumen perimeter size in *perd*-depleted muscles and control muscles.

Quantification of muscle lumen perimeter size, n=20. Muscle lumen perimeter size is maintained constant between 50 and 100 h APF in control muscles. Muscle lumen perimeter size is similar in *perd*-depleted muscles to the controls at 50 h APF. At 100 h APF muscle lumen perimeter size is slightly but significantly bigger in *perd*-depleted muscles compared to the controls. Bars indicate the average, and error bars indicate the standard deviation. ns indicates a non-significant p value. *** indicates a p value < 0.001.

Supplementary Fig. 3. Talin localizes properly in *perd*-depleted muscles.

(A-C) Confocal micrographs of control **(A, B)** and *perd*-depleted muscles **(C)**. **(A)** In control muscles, Talin (green) colocalizes with Zormin (red) at the Z-bands

(arrowhead). In addition, Talin is localized at the muscle attachment site (arrow). **(B, C)** Actin labelling with Rhodamine-Phalloidin is used to see the myofibrils. **(C)** Talin localization is not affected in *perd*-depleted muscles. Scale bars: 5 μm.

Supplementary Fig. 4. *perd* and *mys* interact genetically during adult myogenesis.

(A-C') Confocal micrographs of muscles stained for Rhodamine-Phalloidin. **(A, A')** Muscles expressing the *perd* RNAi JF01159 are misoriented (arrow in **A'**). **(B, B')** The expression of *mys* RNAi presents misoriented muscles and muscle detachment (arrow and arrowhead, respectively in **B'**). **(C, C')** Co-expression of both RNAi constructs results in a clear increase in the number of both misoriented and detached muscles compared to the single expression of each RNAi. **(D)** Quantification of the percentage of misoriented muscles per hemisegment, n=9. **(E)** Quantification of the percentage of detached muscles per hemisegment, n=9. A3, A4 and A5 indicate the corresponding abdominal segments. PLM: Persistent Larval Muscle. Scale bars: 20 μm. Bars indicate the average, and the error bars indicate the standard deviation. Bars labelled with different letters indicate statistically significant differences.

Supplementary Movie 1. Imaging of adult myogenesis in control pupae.

Mef2-GAL4 > UAS-mCD8-GFP. Larval, adult muscles, and hemocytes are labelled with mCD8-GFP. In the first frame, the red dashed line indicates the adult muscle precursors. Asterisks label the persistent larval muscles. Note the orientation of adult muscles along the anterior-posterior axis and a clear separation of individual muscle fibres. Movie starts at 20h APF. Segment A2 shown. Image 297 μm^2. n=7 pupae.

Supplementary Movie 2. Imaging of adult myogenesis in *perd*-depleted pupae.

Mef2-GAL4 > UAS-mCD8-GFP, UAS-*perd* 106680. Larval, adult muscles, and hemocytes are labelled with mCD8-GFP. In the first frame, the red dashed line indicates the adult muscles. Note some muscle fibres that are misoriented (white arrow) and that fibres do not separate properly. A cyan asterisk labels a persistent larval muscle, which shows a rounded phenotype compared to the elongated wild-

type morphology. Movie starts at 20h APF. Segment A2 shown. Image 294 μm^2. n=7 pupae.

Supplementary Figure 1

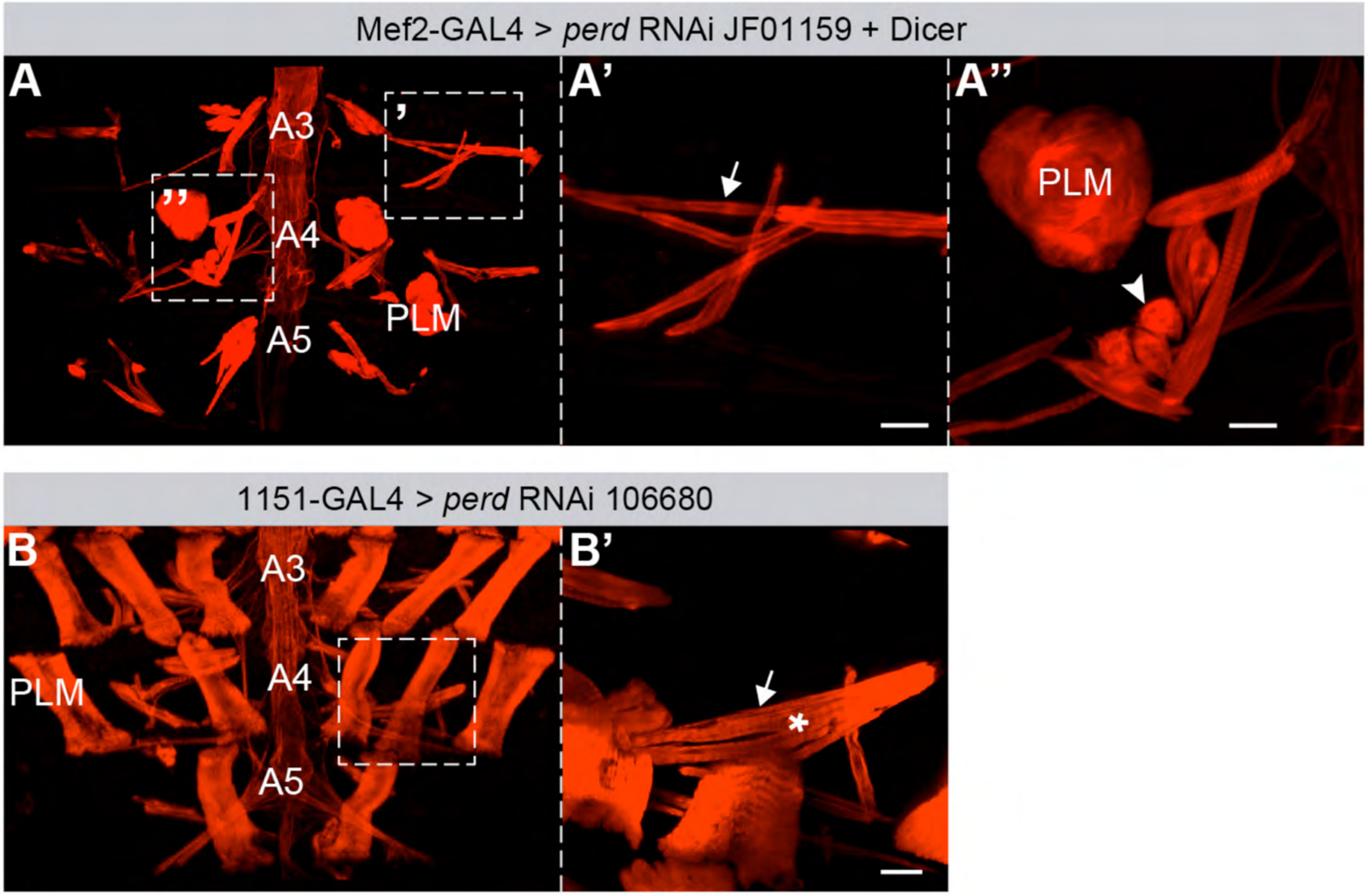

Supplementary figure 2

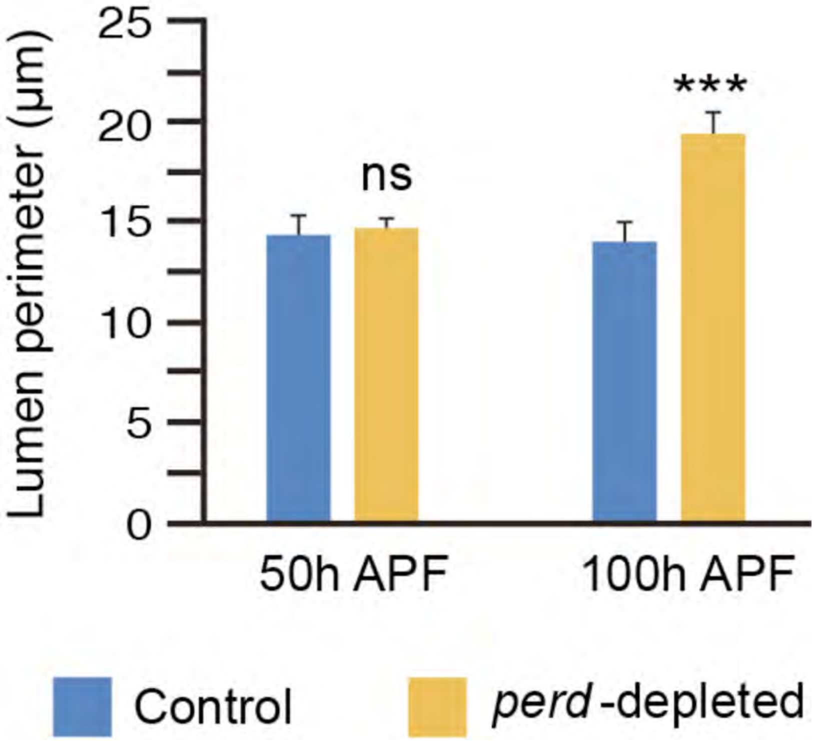

Supplementary Figure 3

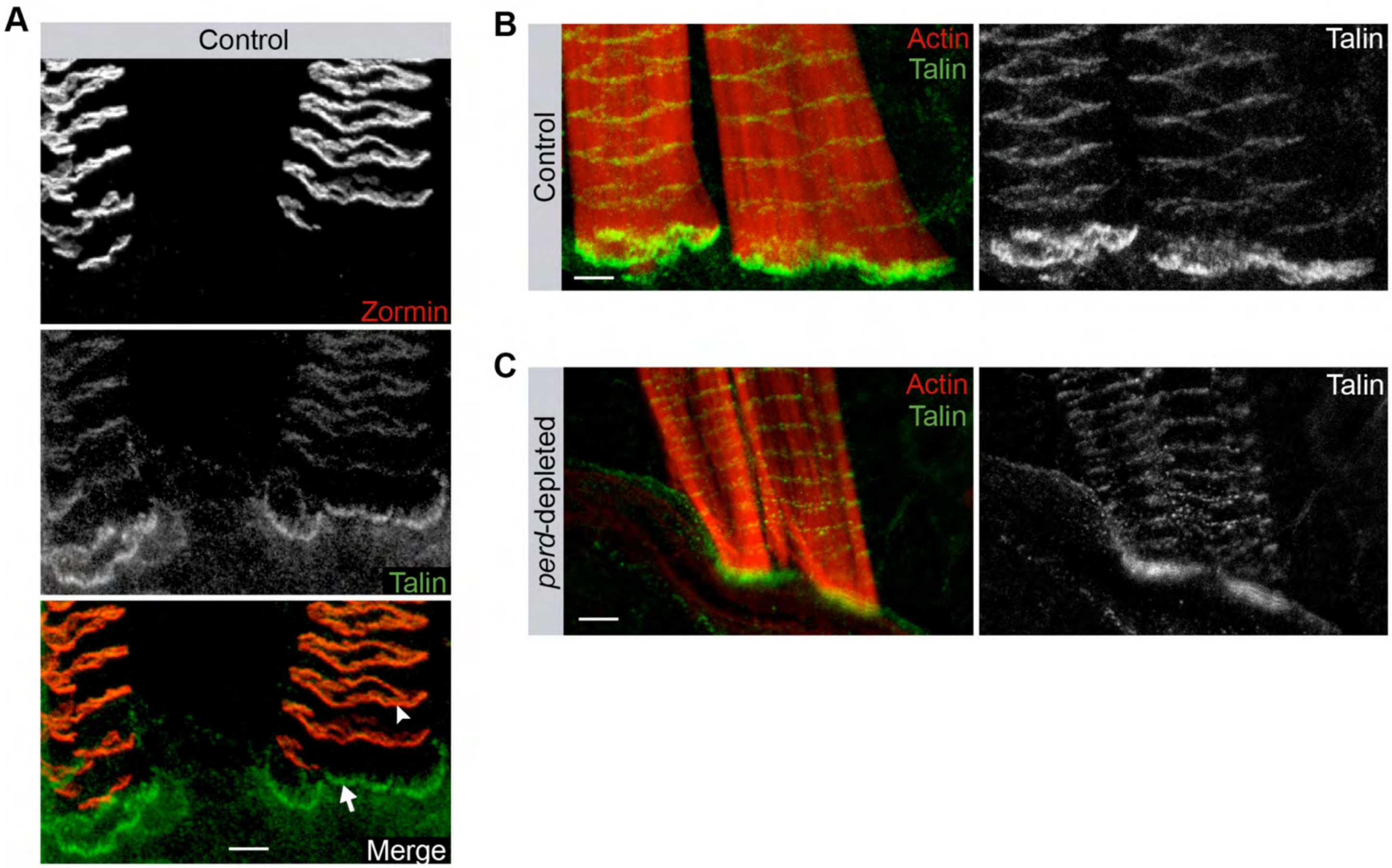

Supplementary figure 4

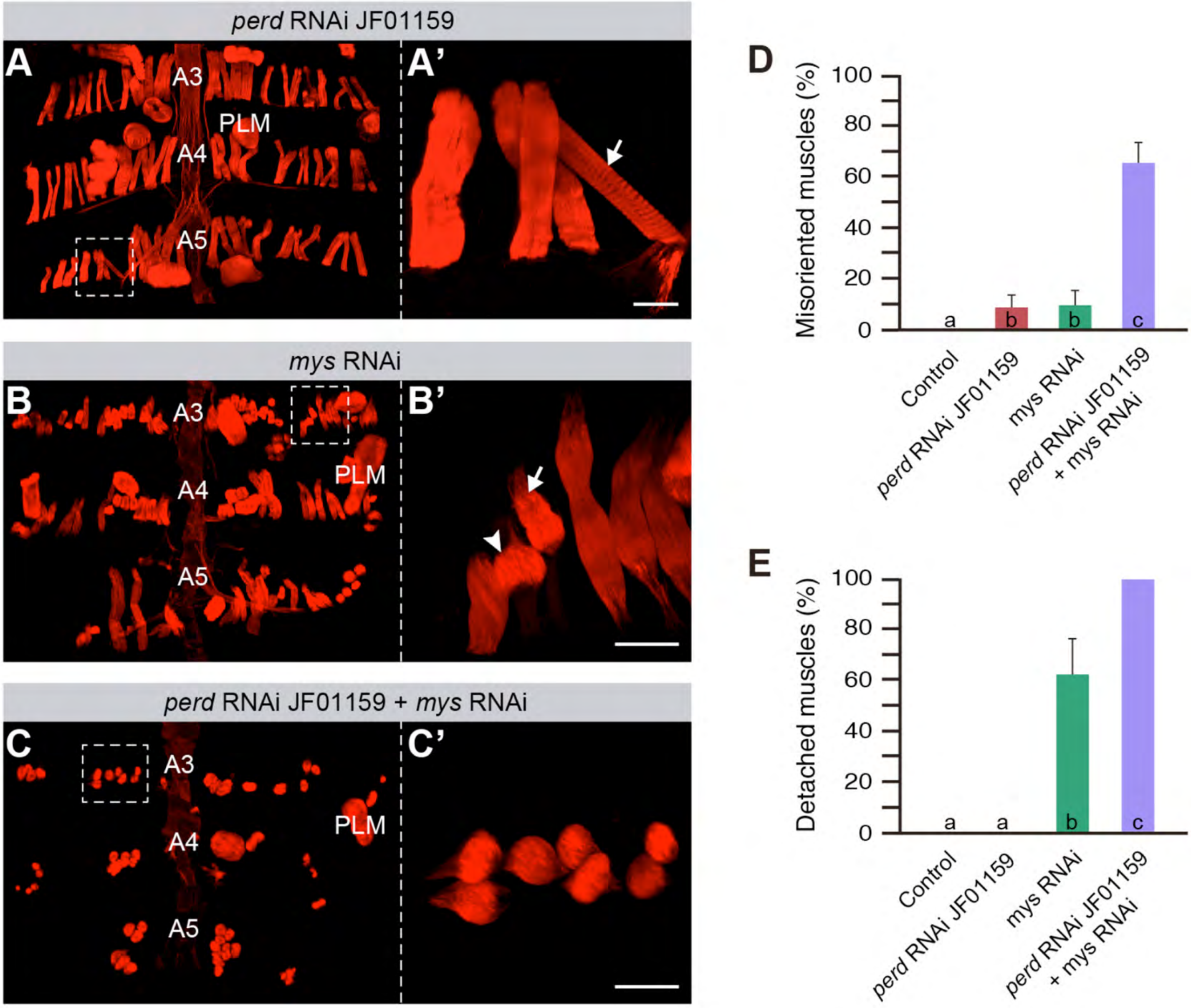

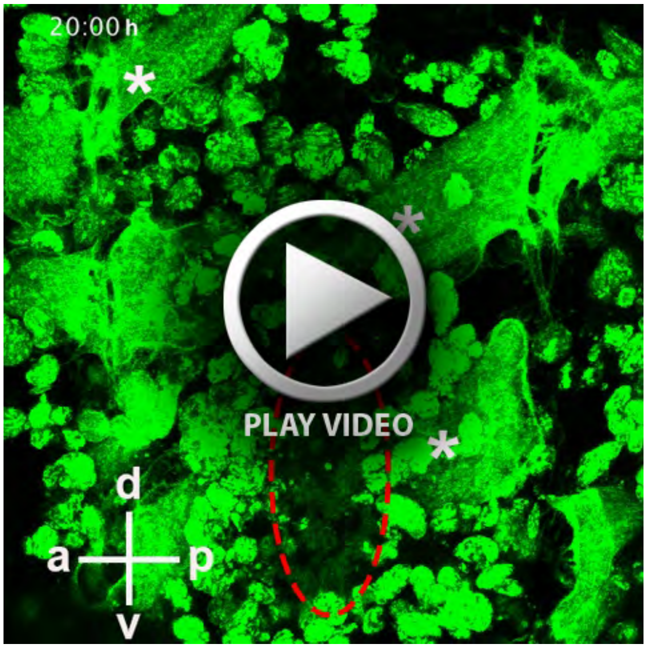

Movie 1.

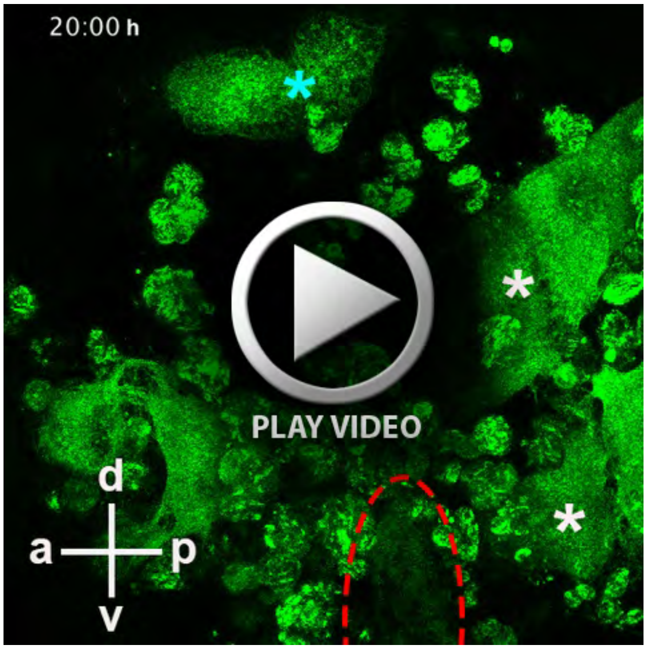

Movie 2.
